# Corynebacterium minutissimum as a Rare Cause of Tibial Osteomyelitis: A Case Report and Literature Review

**DOI:** 10.7759/cureus.75885

**Published:** 2024-12-17

**Authors:** Ayub Ansari, Dania Shoaib, Maher Asfour, Xiyue Wang, Pooja Sharma

**Affiliations:** 1 Internal Medicine, Kansas City University of Medicine and Biosciences, Kansas City, USA; 2 Internal Medicine, O'Connor Hospital, Santa Clara, USA; 3 Obstetrics and Gynecology, The Chinese University of Hong Kong, Hong Kong, CHN

**Keywords:** atypical cultures in orthopedics, corynebacterium species, orthopedic infection, orthopedic surgery, osteomyelitis treatment, tibial infection, tibial osteomyelitis

## Abstract

Osteomyelitis is commonly caused by pathogens like *Staphylococcus aureus*, but rare organisms such as *Corynebacterium minutissimum*, typically associated with superficial skin infections, can also be implicated. Recognizing these atypical pathogens presents diagnostic and therapeutic challenges, especially in the presence of orthopedic hardware. We conducted a literature review yielding 25 studies and encompassing 797 patient cases, which highlights the emerging role of *Corynebacterium* species in osteomyelitis, particularly following trauma or surgical interventions. A 72-year-old man with a history of chronic right tibia and fibula fractures from a motor vehicle accident presented with progressively worsening leg pain over six months. Imaging revealed hardware failure and new fractures. Surgical intervention involved hardware removal, osteotomy, and placement of an external fixator. Intraoperative cultures eventually grew *C. minutissimum* which was resistant to ceftriaxone but sensitive to doxycycline. His antibiotic regimen was switched from intravenous cefazolin to oral doxycycline, leading to gradual pain improvement and stable clinical status. This case adds to the growing body of literature on *C. minutissimum* as a rare but significant cause of osteomyelitis, particularly in patients with orthopedic hardware. Our literature review emphasizes the need for clinicians to be vigilant for *Corynebacterium* species in cases of osteomyelitis unresponsive to standard treatments. Early recognition and targeted antimicrobial therapy guided by susceptibility testing are crucial for successful outcomes in managing atypical bone infections.

## Introduction

Osteomyelitis is a serious and often debilitating bone infection commonly caused by pathogens such as *Staphylococcus aureus* and *Streptococcus* species, particularly after an injury or a surgical procedure. However, less common organisms like *Corynebacterium minutissimum*, which are usually linked with superficial skin infections like erythrasma, are now being recognized as rare but important causes of osteomyelitis [1,2]. The role of *C. minutissimum* in bone infections poses unique challenges for diagnosis and treatment. Its capacity to form biofilms and invade bone cells makes infection clearance difficult, especially in the presence of orthopedic hardware [1]. Due to this, chronic osteomyelitis caused by *Corynebacterium* species often leads to poor clinical outcomes if not identified and treated promptly.

The clinical standard for treating such cases typically involves surgical debridement followed by targeted antimicrobial therapy guided by susceptibility testing [3]. However, choosing the right antibiotics can be difficult due to the organism's variable resistance profiles [4]. In this report, we present a rare case of tibial osteomyelitis caused by *C. minutissimum*. This case highlights the importance of recognizing atypical pathogens in bone infections and emphasizes the challenges in both diagnosis and treatment. After encountering this rare and clinically significant infection, we carried out a comprehensive literature review. Our aim is to evaluate the current understanding of the pathogenesis, diagnosis, and treatment of osteomyelitis caused by *Corynebacterium*. By sharing our findings and insights from this case and literature review, we hope to raise awareness among clinicians and provide guidance for effectively managing similar cases in the future.

## Case presentation

A 72-year-old man with a past medical history of fractures to his right tibia and fibula in a car accident 10 years ago presented with progressively worsening right lower limb pain over the past six months. His original fractures were complicated by chronic non-union and recurrent infections, leading to multiple surgical interventions over the past decade. These procedures included hardware placement, external fixation, and bone grafting. Despite all these efforts, the patient was still unable to bear weight on his right leg and relied on a wheelchair for mobility. Upon admission, the patient reported severe, constant right leg pain localized to the lateral aspect of his shin which worsened with movement. He did not have any fever, chills, or other systemic symptoms. On examination, there was tenderness over the right tibia and fibula, with no signs of erythema, warmth, or swelling. The surgical sites were healed, and there were no open wounds or drainage. A neurovascular exam of the right lower limb showed no abnormalities.

Imaging studies were performed to determine the cause of his pain. X-rays of the right lower limb showed a fractured surgical plate and a new fracture of the fibula. The images suggested hardware failure and possible underlying bone pathology. Based on these findings, the orthopedic team decided to proceed with surgical intervention, including removal of the hardware, osteotomy, and placement of an external fixator. During the surgery, intraoperative samples were collected for culture and sensitivity testing. While there were no signs of obvious pus or necrotic tissue, there was clear evidence of chronic inflammation. After the procedure, the patient was empirically started on intravenous cefazolin (Ancef) at a dose of 2 grams every eight hours. This antibiotic was selected to target common organisms associated with osteomyelitis.

On the seventh day after surgery, the patient was moved to a skilled nursing facility for rehabilitation. At the time of transfer, he was still experiencing significant pain, rated as an 8 out of 10, in the lateral shin that worsened with any attempt to move. Despite being prescribed pain medication, including hydrocodone-acetaminophen (Norco), his pain remained poorly controlled. Physical therapy was initiated but had limited progress, as he was reluctant to participate due to the severity of his pain. On the 11th day after surgery, the microbiology lab reported that the wound cultures had grown *C. minutissimum*, a rare cause of osteomyelitis. The bacteria demonstrated resistance to ceftriaxone, but was sensitive to doxycycline, trimethoprim-sulfamethoxazole, vancomycin, and linezolid. Based on these findings, the infectious disease team was brought in, and his antibiotics were switched from intravenous cefazolin to oral doxycycline.

In the following weeks, the patient reported a gradual decrease in pain, with his self-reported scores decreasing from an 8 out of 10 to a 4 out of 10. He remained afebrile, with stable vital signs and a good appetite. He had no new concerns. Although his pain had reduced, his engagement in physical therapy remained limited. While he expressed a strong desire to regain mobility, he was hesitant to participate fully in rehabilitation exercises due to lingering discomfort. During this time, lab tests showed no significant abnormalities. Routine blood work, including complete blood count and basic metabolic panel, were within normal limits. Inflammatory markers remained stable, with no notable elevations. Follow-up imaging studies showed no signs of worsening bone infection or complications related to the new hardware. The external fixator was properly positioned with no signs of loosening or infection at the pin sites. The surgical wounds were clean and healing well without signs of erythema, warmth, or discharge.

Three weeks after surgery, the patient remained on oral doxycycline as directed by the infectious disease team. Regular follow-up appointments were scheduled with both the orthopedic and infectious disease teams to track his recovery. Despite continued efforts from the rehabilitation team, his progress in physical therapy remained slow. His pain management plan was reviewed, and adjustments were made to his medications to encourage better participation in therapy.

## Discussion

*C. minutissimum* is a gram-positive, non-spore-forming, aerobic, or facultatively anaerobic bacillus commonly associated with superficial skin infections such as erythrasma, characterized by reddish-brown macular patches. Extracutaneous infections caused by *C. minutissimum* are rare but have been documented, including cases of bacteremia, meningitis, pyelonephritis, and endocarditis [5-7]. However, osteomyelitis due to *C. minutissimum* is exceedingly uncommon, making this case significant as it demonstrates the pathogen's potential to cause deep-seated bone infections.

**Figure 1:**
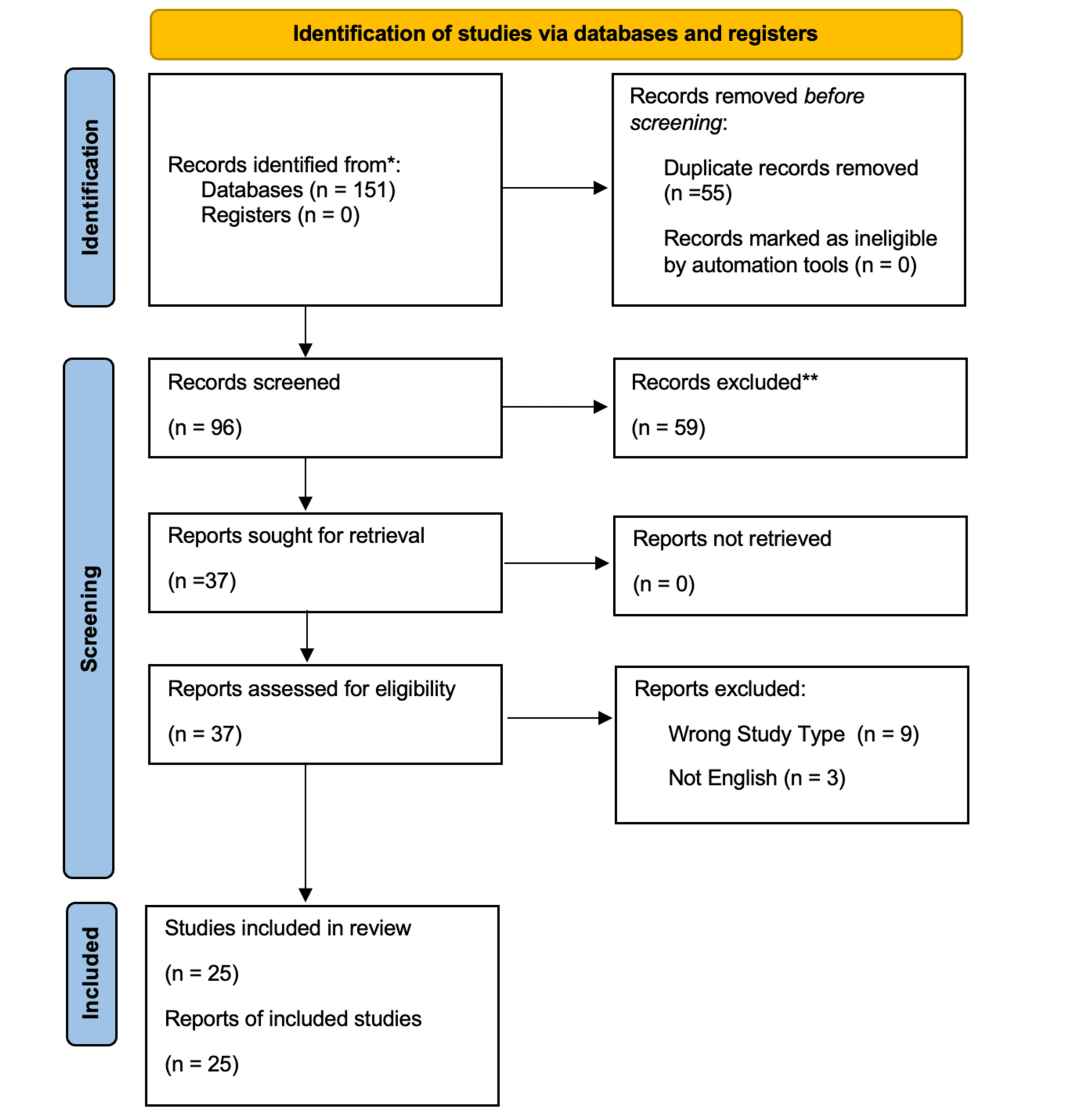
The PRISMA workflow diagram *The number of records identified from the database and register searches. **Any records excluded were excluded by a human; no automation tools were used. PRISMA: Preferred Reporting Items for Systematic Reviews and Meta-Analyses

To better understand the role of *Corynebacterium* species in osteomyelitis, we conducted a literature review following the Preferred Reporting Items for Systematic Reviews and Meta-Analyses (PRISMA) guidelines. Our search strategy included conducting searches on PubMed and Embase databases, using the Boolean operators "AND" and "OR" to combine keywords such as "Corynebacterium", "Corynebacterium osteomyelitis", "Corynebacterium bone infection", "Corynebacterium species", "Corynebacterium minutissimum", "Corynebacterium jeikeium", and "Corynebacterium striatum" with terms like "osteomyelitis", "bone infection", and "orthopedic infection". After screening titles, abstracts, and full texts according to predefined inclusion and exclusion criteria, we identified 25 studies encompassing a total of 797 patient cases (Figure 1). 

We performed a qualitative risk of bias assessment for each included study. Since the included articles were case reports, small case series, or retrospective cohorts without control groups, the effect measure consisted of qualitative outcomes such as clinical improvement, radiographic resolution, or healing. Two independent reviewers (AA and DS) assessed each study's risk of bias based on participant selection, clarity of interventions and outcomes, adequacy of follow-up, and potential sources of bias. Each domain was judged as "low", "moderate", or "high" risk of bias, and any discrepancies were resolved by consensus. Most studies were categorized as having a "moderate" risk of bias due to limited sample sizes, lack of control groups, and variable reporting clarity. Two studies demonstrated "high" risk in certain domains due to incomplete follow-up or persistent disease. This detailed, study-by-study appraisal is summarized in the risk of bias assessment (Table 1). By transparently outlining these parameters, we address the PRISMA recommendations and provide a clearer understanding of the evidence quality guiding our conclusions.

**Table 1:** Risk of bias assessment for included studies evaluating Corynebacterium spp. osteomyelitis L: low risk of bias; M: moderate risk of bias; H: high risk of bias; N: number of patients; CRP: C-reactive protein; ESR: erythrocyte sedimentation rate; IV: intravenous; NR: not reported; RA: rheumatoid arthritis; TKA: total knee arthroplasty; PMMA: polymethyl methacrylate; OA: osteoarthritis; ROM: range of motion

Study	Design (case/series/cohort)	Effect measure (qualitative improvement/resolution)	Participant selection (L/M/H)	Interventions and outcomes (L/M/H)	Follow-up (L/M/H)	Reporting and bias (L/M/H)	Overall risk of bias
Kong et al. [8]	Case (N=1)	Qualitative (healed infection, functional recovery)	Mod (single patient, no criteria)	Mod (treatments described, limited detail)	Mod (36 months follow-up adequate but single case)	Mod (no control, possible publication bias)	Moderate
Sasaki et al. [9]	Case (N=1)	Qualitative (callus formation, return to work)	Mod (single patient, diabetes)	Mod (clear interventions, outcomes defined)	Low (36 months sufficient)	Mod (no control group, selective reporting possible)	Moderate
Atalay et al. [10]	Case (N=1)	Qualitative (radiologic resolution at 10 months)	Mod (no selection criteria)	Mod (therapy described, outcome defined)	Low (10 months acceptable)	Mod (single scenario, no comparator)	Moderate
Morrey et al. [11]	Series (N=3)	Qualitative (clinical improvement, recurrence noted)	Mod (three patients, no uniform criteria)	Mod (interventions/outcomes described per case)	Mod (15-20 months follow-up adequate, one limited by sarcoma)	Mod (small series, potential selective reporting)	Moderate
Sharma et al. [12]	Case (N=1)	Qualitative (clinical and radiological improvement)	Mod (one patient, severe comorbidity)	Mod (intervention noted, outcomes clear but brief)	Mod (~1 month short but some improvement)	Mod (no control, short follow-up)	Moderate
Verma and Kravitz [13]	Case (N=1)	Qualitative (successful treatment, discharge)	Mod (complex comorbidities)	Mod (basic clarity, limited methodology)	Mod (NR exact beyond discharge)	Mod (no long-term data, single case)	Moderate
Wong et al. [14]	Case (N=1)	Qualitative (clinical improvement, normalized markers)	Mod (no defined criteria, single patient)	Mod (treatment steps reported, outcomes known)	Mod (NR exact follow-up duration)	Mod (single case, no control)	Moderate
Boc and Martone [15]	Case (N=1)	Qualitative (wound improvement)	Mod (single patient, limited detail)	Mod (interventions known, outcomes short-term)	High (lost to follow-up, final outcome unclear)	High (incomplete outcome assessment)	High
Bayram et al. [16]	Case (N=1)	Qualitative (clinical resolution, normal CRP/ESR)	Mod (pediatric, no selection criteria)	Mod (clear interventions/outcomes)	Low (24 months follow-up good)	Mod (single case)	Moderate
Ceilley [17]	Case (N=1)	Qualitative (infection resolution)	Mod (one patient, no selection criteria)	Mod (antibiotics partly unspecified, outcomes known)	Mod (12 months follow-up acceptable)	Mod (no control, basic data)	Moderate
Moyad [18]	Case (N=1)	Qualitative (no recurrence, improved ROM)	Mod (one patient, OA)	Low (very clear interventions, outcomes well-defined)	Low (18 months excellent)	Mod (single case, no control)	Moderate
Weller et al. [19]	Series (N=2)	Qualitative (successful resolution)	Mod (two patients, no broad criteria)	Mod (interventions and outcomes described)	Low (>12 months follow-up sufficient)	Mod (small series, possible bias)	Moderate
Poilane et al. [20]	Case (N=1)	Qualitative (rapid recovery)	Mod (child patient, no criteria)	Mod (treatment steps given, outcomes brief)	Mod (NR long-term follow-up)	Mod (no long-term data)	Moderate
Fernández-Ayala et al. [21]	Case (N=1)	Qualitative (complete recovery, improved imaging)	Mod (no selection criteria)	Mod (interventions stated, outcomes clear)	Low (12 months follow-up adequate)	Mod (single case)	Moderate
Krish et al. [22]	Case (N=1)	Qualitative (clinical and imaging improvement)	Mod (unclear selection)	Mod (detailed antibiotic regimen, outcome defined)	Low (12 months follow-up good)	Mod (single case, no control)	Moderate
Chomarat et al. [23]	Case (N=1)	Qualitative (improvement at one year)	Mod (single patient, complex history)	Mod (antibiotics stated, outcome defined)	Low (12 months follow-up)	Mod (no control, possible bias)	Moderate
Ordóñez-Palau et al. [24]	Case (N=1)	Qualitative (complete resolution post-surgery)	Mod (RA patient, unique scenario)	Mod (no postoperative antibiotics, outcome clear)	Low (12 months follow-up adequate)	Mod (single patient)	Moderate
Munian et al. [25]	Case (N=1)	Qualitative (complete resolution, consolidation)	Mod (single patient, complex interventions)	Mod (clear steps, outcome known)	Low (12 months good)	Mod (no control)	Moderate
Farnam et al. [26]	Case (N=1)	Qualitative (partial resolution, multifocal persistence)	Mod (immunodeficiency)	Mod (outcomes complex, interventions known)	High (NR exact long-term outcome, persistent issues)	High (persistent disease, unclear endpoint)	High
Chauvelot et al. [1]	Retro. cohort (N=51)	Qualitative (healing rates, infection persistence)	Mod (selected population, no randomization)	Mod (outcomes reported but heterogeneous)	Mod (~14 months median follow-up)	Mod (no control, retrospective)	Moderate
Virgilio et al. [27]	Retro. cohort (N=50)	Qualitative (healing vs. poor healing)	Mod (diabetic, vascular compromise)	Mod (clearly states healing vs. not, interventions known)	Mod (12 months follow-up)	Mod (no control, retrospective)	Moderate
Rizvi et al. [28]	Retro. cohort (N=576)	Qualitative (infection presence/absence)	Mod (large, heterogeneous sample)	Mod (broad data, outcomes as presence/absence)	Mod (NR exact follow-up, implied)	Mod (no control, retrospective)	Moderate
Tocco et al. [29]	Retro. cohort (N=70)	Qualitative (healing rates, relapses)	Mod (post-cardiac surgery patients)	Mod (varied interventions, outcomes defined)	Mod (12 months follow-up)	Mod (no control, retrospective)	Moderate
Johani et al. [30]	Retro. cohort (N=20)	Qualitative (healing vs. persistence)	Mod (diabetic foot ulcers)	Mod (biofilm noted, outcomes stated)	Mod (NR exact follow-up)	Mod (no control, retrospective)	Moderate
Trivedi and Merchant [31]	Retro. cohort (N=7)	Qualitative ("cure" as wound granulation, symptom resolution)	Mod (small cohort, unclear selection)	Mod (definition of cure stated, interventions noted)	Mod (1-2 months short but stated)	Mod (no control, retrospective)	Moderate

Data extracted from this search are presented in Table 2. The literature on *Corynebacterium* species causing osteomyelitis is limited but growing. In our review of these 25 studies involving osteomyelitis caused by *Corynebacterium *species, several key insights emerged. The most commonly reported species were *Corynebacterium striatum*, *Corynebacterium jeikeium*, *Corynebacterium amycolatum*, *Corynebacterium diphtheriae*, *Corynebacterium pseudodiphtheriticum*, and *Corynebacterium xerosis*. Chauvelot et al. reported that 74.5% of their cases had prior surgeries or trauma, highlighting the role of surgical interventions in the pathogenesis of *Corynebacterium *osteomyelitis [1]. Similarly, Weller et al. described cases where *C. jeikeium *was isolated following prosthetic hip replacement complications, suggesting that orthopedic hardware implantation can be a predisposing factor [19].

**Table 2:** Clinical, microbiological, and management data from 25 studies (N=797 patients) involving Corynebacterium spp. osteomyelitis CRP: C-reactive protein; WBC: white blood cell; ESR: erythrocyte sedimentation rate; IV: intravenous; VAC: vacuum-assisted closure; TKA: total knee arthroplasty; PMMA: polymethyl methacrylate; IMT: induced membrane technique; NR: not reported

Study	Age (years), gender	Comorbidities	Immune status	Pathogenesis	Clinical presentation	Site of osteomyelitis	Organism(s)	Presumed mode of infection	Treatment: pharmacological	Treatment: surgical	Outcome	Follow-up (months)
Kong et al. [8]	45, male	Dermatophytosis (recurrent)	Immunocompromised	Relapsed polymicrobial osteomyelitis	Pain, redness, swelling, pruritus, and heating over the left tibia; chronic dermatophytosis on the left heel	Left tibia	*Corynebacterium *spp.; *Staphylococcus aureus*	Traumatic inoculation with a rusty object at age 10; relapse triggered by fungal dermatophytosis	Ceftizoxime for 30 days; adjusted to piperacillin/tazobactam for 35 days after sensitivity testing	Debridement and VAC therapy performed three times	Healed; recurrent dermatophytosis correlated with osteomyelitis relapses; patient functionally recovered with intermittent symptoms	36
Sasaki et al. [9]	46, male	Diabetes mellitus	NR	Chronic osteomyelitis with extensive femoral diaphysis defect, complicated by subtrochanteric fracture during the waiting period for surgery	Persistent right thigh pain; restricted hip and knee motion; localized heat; elevated inflammatory markers (CRP: 20.8 mg/dL, WBC: 18,800/μL)	Right femoral diaphysis	*Corynebacterium *spp. (species unspecified)	NR	Linezolid 600 mg twice daily for 3 days; switched to daptomycin 8 mg/kg once daily for 4 weeks	Two-stage IMT: Stage 1: Debridement and placement of vancomycin-impregnated PMMA spacer. Stage 2: Grafting with autologous cancellous bone and β-tricalcium phosphate	Complete healing with callus formation in 3 out of 4 cortices; patient returned to work 6 months after stage 2 surgery	36
Atalay et al. [10]	55, male	Chronic renal failure	Immunocompromised	Spontaneous *Corynebacterium diphtheriae* discitis with associated osteomyelitis	Acute low back pain; weakness in the anterior tibialis and extensor hallucis longus; hypoesthesia in L4-L5 dermatomes; elevated CRP (73 mg/L) and ESR (70 mm/h)	L5-S1 vertebral bodies (discitis and osteomyelitis)	Corynebacterium diphtheriae	Hematogenous spread, likely through skin breaks caused by pruritus or dialysis-related procedures (e.g., catheterization)	Vancomycin and ceftriaxone IV for 4 weeks; added rifampicin; continued triple therapy for an additional 4 weeks (total duration: 8 weeks)	L5-S1 discectomy with nerve root decompression	Significant improvement after 8 weeks of antibiotic therapy; follow-up MRI at 10 months showed near-complete resolution with sclerosis at L5-S1	10
Morrey et al. [11]	Case 1: 65, female	Case 1: None specified	Case 1: Immunocompetent	Case 1: Postoperative infection after hip fixation surgery	Case 1: Painful left hip; high sedimentation rate (100 mm/h)	Case 1: Left hip joint	Case 1: *Propionibacterium acnes*	Case 1: Surgical introduction during surgery (iatrogenic)	Case 1: Methicillin IV; switched to penicillin G IV; followed by oral penicillin for 3 months	Case 1: Removal of implants, excision of granulation tissue, Girdlestone arthroplasty; later underwent total hip arthroplasty	Case 1: Excellent motion without pain 15 months post-surgery; sedimentation rate normalized	Case 1: 15
	Case 2: 71, male	Case 2: Chronic idiopathic urticaria; history of myocardial infarction; prednisone use; prior elbow surgery	Case 2: Immunocompromised	Case 2: Recurrent infection following initial elbow trauma and surgeries	Case 2: Sinus tract with drainage; limited elbow movement; severe deformity	Case 2: Elbow joint (distal humerus and proximal ulna)	Case 2: *Staphylococcus epidermidis*, *Propionibacterium acnes*, and *Corynebacterium *spp.	Case 2: Repeated surgeries leading to compromised local tissue	Case 2: Clindamycin IV for 1 week; then oral clindamycin for 4 weeks	Case 2: Resection of affected bones, debridement, and stabilization using Steinmann pins	Case 2: Healed without recurrence 20 months post-surgery	Case 2: 20
	Case 3: 50, male	Case 3: Severe burn scars; chronic osteomyelitis since adolescence	Case 3: Immunocompetent	Case 3: Chronic infection due to burn-related contractures and recurrent ulcers	Case 3: Draining sinuses; ankylosed elbow in 90° flexion; weak grip; pain	Case 3: Elbow joint (proximal radius and ulna; distal humerus)	Case 3: Group D *Streptococcus*, *Corynebacterium *spp.; later *Pseudomonas aeruginosa*	Case 3: Chronic soft tissue breakdown and multiple surgical interventions	Case 3: Vancomycin initially; adjusted to erythromycin, gentamicin, carbenicillin, and penicillin after desensitization	Case 3: Multiple debridements, resection of infected bone, and skin grafting	Case 3: Recurrence of infection; diagnosed with epithelioid sarcoma; underwent shoulder disarticulation	Case 3: Limited follow-up due to sarcoma diagnosis
Sharma et al. [12]	50, female	Diabetes mellitus (uncontrolled); HbA1c 9.2%	Immunocompromised	Emphysematous osteomyelitis caused by *Escherichia coli* and *Corynebacterium amycolatum*, involving intraosseous gas formation	Chronic low back pain radiating down the left lower limb; numbness, weakness, sensory loss; intermittent low-grade fever; neurological deficits below L1	Spine (L5-S1 vertebral discitis), pelvis (sacrum and sacroiliac joint), and iliopsoas muscle	*Escherichia coli*; *Corynebacterium amycolatum*	Hematogenous spread from a previous infection, possibly originating from a bedsore	Initial: Piperacillin-tazobactam IV for 7 days; clindamycin added for anaerobic coverage. Adjusted to: Meropenem 1 g IV three times daily for a total of 6 weeks	Surgical drainage of pus from the left iliopsoas area under CT guidance	Significant improvement in renal function and sensorium; clinical and radiological improvement following appropriate antibiotic treatment	~1
Verma and Kravitz [13]	69, female	Advanced rheumatoid arthritis (28 years); fibromyalgia; chronic fatigue syndrome; hypertension; complete heart block	Immunocompromised	Opportunistic infection facilitated by long-term immunosuppression and chronic antibiotic use	Shortness of breath; productive cough; fever; right-sided abdominal pain; chronic ulceration with exposed bone in the second right toe	Second toe of the right foot	Corynebacterium striatum	Contiguous spread from chronic toe ulceration to underlying bone; exacerbated by systemic immunosuppression	Initially treated with imipenem; switched to vancomycin IV for 2 weeks based on sensitivity results	No surgical intervention mentioned	Successfully treated with surgical intervention and vancomycin; discharged to a nursing home on postoperative day 18	NR
Wong et al. [14]	69, male	Basal cell carcinoma (recent excision); partial knee joint replacement	NR	Opportunistic infection following trauma (fall causing pubic fracture) and possible contamination from basal cell carcinoma excision	Persistent left hip pain; tenderness around the pubic symphysis; elevated inflammatory markers (CRP: 288 mg/L, ESR: 23 mm/h)	Left symphysis pubis	Corynebacterium accolens	Likely contiguous spread from trauma-induced fracture or wound contamination during basal cell carcinoma excision	Initial: Empirical flucloxacillin. Adjusted to: Cefazolin IV for 4 weeks; followed by oral amoxicillin for 4 weeks	Ultrasound-guided aspiration of a soft tissue abscess; no debridement or invasive surgery performed	Significant clinical improvement; normalization of inflammatory markers (CRP: 6 mg/L, ESR: 9 mm/h)	NR
Boc and Martone [15]	54, male	Insulin-dependent diabetes mellitus (well-controlled); history of mid-forearm amputation due to prior hand infection	NR	Contiguous spread from a chronic wound and abscess on the fifth submetatarsal area	Painful callus (tyloma) on the fifth submetatarsal; swelling; erythema; draining purulent discharge from a perforated wound	Right fifth metatarsal	Corynebacterium jeikeium	Chronic wound infection with contiguous spread to underlying bone	Initial: Empirical antibiotics. Adjusted to: Vancomycin IV; duration unspecified but continued during hospitalization	Aggressive soft tissue debridement and partial amputation of the fifth metatarsal	Significant wound improvement observed after 7 days; patient discharged against medical advice; long-term outcome unknown	Lost to follow-up
Bayram et al. [16]	4, male	None	Immunocompetent	Infection due to trauma from a wood splinter leading to contiguous spread	1-month history of ankle pain, swelling, erythema, and limping; localized edema and rubor	Distal fibula (metadiaphyseal region)	Corynebacterium striatum	Traumatic inoculation from a wood splinter injury without a visible skin wound	Vancomycin and cefazolin IV for 6 weeks	Open biopsy and curettage of the distal fibula to remove infected material	Complete clinical resolution; normalization of blood markers (CRP: 0.5 mg/L, ESR: 11 mm/h) at follow-up; no complications noted	24
Ceilley [17]	62, male	Diabetes mellitus; peripheral vascular disease	Immunocompetent	Infection associated with foot ulceration leading to vertebral osteomyelitis	Chronic back pain; symptoms associated with vertebral infection	Vertebrae	Corynebacterium haemolyticum	Hematogenous spread from a foot ulcer	Intravenous antibiotics (details unspecified); followed by oral antibiotics. Total duration: 6 weeks	No surgical intervention mentioned	Resolution of infection; significant clinical improvement	12
Moyad [18]	67, female	Osteoarthritis	Immunocompetent	Chronic osteomyelitis secondary to *Corynebacterium pseudodiphtheriticum* infection following arthroscopic meniscal surgery	Chronic knee pain and stiffness; limited range of motion (20° flexion contracture, maximum flexion of 55°); mild warmth and swelling; absence of redness or effusion	Right knee joint (tibial plateau and lateral femoral condyle)	Corynebacterium pseudodiphtheriticum	Postoperative infection following arthroscopic surgery; delayed diagnosis suggests a smoldering infection developing over time	Penicillin IV for 6 weeks	Two-stage TKA: Stage 1: Radical debridement, synovectomy, and placement of an antibiotic-loaded articulating spacer. Stage 2: Definitive TKA after the normalization of inflammatory markers	No recurrent infection at follow-up; pain-free with prolonged ambulation; improved range of motion (0° extension to 88° flexion); postoperative radiographs showed a properly aligned TKA without evidence of loosening or infection	18
Weller et al. [19]	Case 1: 52, female	Case 1: Complications after prosthetic hip replacement (ectopic bone growth; infected hematoma; dislocation)	Case 1: Immunocompetent	Case 1: Persistent discharging sinuses after hip prosthesis complications leading to osteomyelitis	Case 1: Severe pain; persistent discharging sinuses	Case 1: Pseudo-synovial lining of the right hip joint capsule	Case 1: *Corynebacterium jeikeium*	Case 1: Likely inoculation of the operative site during repeated surgeries	Case 1: Intravenous vancomycin for 6 weeks	Case 1: Removal of the hip prosthesis	Case 1: Successful resolution of infection	Case 1: >12
	Case 2: 44, male	Case 2: Osteoarthritis	Case 2: Immunocompetent	Case 2: Stress fracture of femoral component and formation of a sinus leading to osteomyelitis	Case 2: Formation of a sinus after a stress fracture of the femoral component	Case 2: Right hip joint prosthesis site	Case 2: *Corynebacterium jeikeium*	Case 2: Likely inoculation during repeated surgeries	Case 2: Intravenous vancomycin for 6 weeks	Case 2: Removal of the right hip prosthesis	Case 2: Successful resolution of infection	Case 2: >12
Poilane et al. [20]	3, male	None reported; previously healthy child	Immunocompetent	Osteomyelitis caused by *Corynebacterium diphtheriae *subspecies *mitis*; strain was weakly toxigenic; no portal of entry identified	Pain in the left hip; limping for two days prior to admission; pain on motion, especially inward rotation; afebrile with no systemic symptoms	Left hip (intertrochanteric region and proximal femoral diaphysis)	*Corynebacterium diphtheriae* subspecies *mitis*	Possibly introduced from Morocco by family members; no specific portal of entry identified	Initial: Oral oxacillin and netilmicin. Adjusted to: Oral pristinamycin 50 mg/kg/day for 6 weeks; netilmicin 4.5 mg/kg/day for 7 days	Bone aspirate drainage performed	Rapid and satisfactory recovery; the child regained normal, painless hip motion	NR
Fernández-Ayala et al. [21]	60, male	Recent myocardial infarction; infrainguinal vascular graft; history of smoking; heavy alcohol use	NR	Likely hematogenous spread from community-acquired bacteremia following minor lumbar trauma	Fever, confusion, lumbar back pain, and elevated inflammatory markers (ESR: 80 mm/h)	L3-L4 vertebrae (spondylodiscitis)	Corynebacterium striatum	Transient bacteremia with hematogenous spread following minor trauma to the lower back	Initial: Ciprofloxacin. Adjusted to: Ampicillin 2 g IV every 4 hours plus clindamycin 300 mg IV every 6 hours for 7 days. Continued ampicillin for an additional 5 weeks (total ampicillin duration: 6 weeks)	No surgical intervention required	Complete recovery with the normalization of inflammatory markers and radiological improvement at 12-month follow-up	12
Krish et al. [22]	61, male	Primary autonomic failure (Shy-Drager syndrome); history of lumbar spine surgery	Immunocompetent	Direct inoculation during prior spinal surgery or possible hematogenous spread	Persistent low back and left leg pain; tenderness at L4 vertebral spine; radiological evidence of vertebral destruction (L4-L5)	L4-L5 vertebrae	Corynebacterium xerosis	Likely direct inoculation during prior spinal surgery or secondary hematogenous seeding	Initial: Cefazolin and aztreonam IV. Adjusted to: Ceftriaxone IV for 3 months; oral trimethoprim-sulfamethoxazole for 8 months. Total antibiotic duration: 11 months	No surgical intervention required	Complete clinical resolution; follow-up MRI after 12 months showed significant improvement in vertebral structure	12
Chomarat et al. [23]	60, male	Arteritis of lower extremities; history of femur fractures; multiple septic episodes; frequent hospitalizations	NR	Chronic osteomyelitis likely initiated by in situ contamination during surgical interventions or hematogenous spread	Persistent infection of the femur; recurrent septic episodes; failure of previous treatments	Femur	*Corynebacterium *group D2	Likely in situ contamination during surgical interventions or hematogenous dissemination from colonized skin	Initial: Vancomycin 2 g IV daily for 1 week. Adjusted to: Teicoplanin 400 mg daily for 6 weeks	Muscle flap surgery for closure; no further aggressive debridement required	Significant improvement confirmed by bone scintigraphy at 3 months; absence of symptoms after 1 year	12
Ordóñez-Palau et al. [24]	59, female	Long-standing seropositive rheumatoid arthritis	Immunocompromised	Chronic osteomyelitis caused by direct spread from a plantar ulcer beneath the first metatarsal head	3-month history of pain and a non-healing cutaneous ulcer on the plantar aspect of the right foot; marked erythema and draining fistula on the medial border of the foot; no fever or systemic symptoms	Right foot medial sesamoid bone	*Corynebacterium jeikeium*; *Staphylococcus aureus*	Direct spread secondary to plantar ulceration	No postoperative antibiotic therapy; opted for a "watch and wait" approach	Radical debridement and removal of the infected necrotic sesamoid bone (sesamoidectomy)	Complete resolution of symptoms; asymptomatic and without signs of infection after one year	12
Munian et al. [25]	22, male	None	Immunocompetent	Open fracture of the left distal tibia and fibula due to a motorcycle accident; persistent infection following external fixation and multiple surgeries	Persistent serosal drainage through skin holes; development of pseudoarthrosis; recurrent cellulitis and fistulas over the surgical scar	Left distal tibia and fibula	*Staphylococcus aureus *and *Pseudomonas aeruginosa*; *Corynebacterium jeikeium*	Likely nosocomial infection acquired during repeated surgeries or hospital exposure	Initial treatments: Multiple antibiotic regimens over several months. Final treatment for *Corynebacterium jeikeium*: Teicoplanin 400 mg IV daily for 3 weeks	Initial external fixation of the fracture; bone debridement; placement of Müller plates. Follow-up procedures: Sequential removal of Müller plates and screws with debridement and wound cleansing	Complete resolution of infection; bone consolidation after final treatment	12
Farnam et al. [26]	22, male	Chronic recurrent skin infections since childhood; history of boils and abscesses; suspected underlying immunodeficiency	Immunocompromised	Contiguous spread and hematogenous seeding from recurrent skin infections; possible role of trauma	Chronic swelling, tenderness, and trismus over the mandible; fluctuant masses with multiple skin lesions	Mandible (right body and ramus); multifocal involvement of radius, ulna, tibia, and ankle joints	*Corynebacterium *group JK (L-form)	Chronic hematogenous seeding and local spread from persistent skin infections	Initial: Empirical third-generation cephalosporin. Adjusted to: Tobramycin, rifampin, clindamycin, and erythromycin. Hyperbaric oxygen therapy included; antibiotic treatment lasted several months	En bloc resection of the affected mandible (preserving the condyle); multiple prior debridements	Mandibular osteomyelitis resolved post-resection; however, multifocal osteomyelitis persisted; recurrent skin infections continued	NR
Chauvelot et al. [1]	Median: 54.2 (44.2-68.8); N=51; majority male (70.6%)	Frequent comorbidities included prior surgeries, trauma, or orthopedic device-related infections (74.5%)	NR	Post-traumatic or postoperative inoculation of *Corynebacterium* species, typically associated with orthopedic implants	Chronic infections (88.2%), presenting with pain, swelling, sinus tract formation, and elevated inflammatory markers (e.g., CRP)	Osteomyelitis associated with orthopedic devices (prosthetic joints, osteosynthesis devices)	*Corynebacterium striatum *(37.5%), *Corynebacterium tuberculostearicum* (12.5%), *Corynebacterium simulans *(10.4%), *Corynebacterium jeikeium *(8.3%), *Corynebacterium minutissimum *(8.3%)	NR	Antibiotics varied by species: *Corynebacterium striatum*: Vancomycin or daptomycin, often combined with rifampicin. *Corynebacterium tuberculostearicum*: Linezolid or beta-lactams (e.g., cefazolin). *Corynebacterium simulans*: Beta-lactams or vancomycin. *Corynebacterium jeikeium*: Vancomycin or daptomycin. *Corynebacterium minutissimum*: Linezolid or beta-lactams. Median total antibiotic duration: 18.1 weeks (IQR 13.1-29.3); median IV therapy duration: 14.1 weeks	Surgery performed in 92.2% of cases; optimal surgical management achieved in 76.5%	Treatment failure rate of 39.2%; chronicity and inadequate surgical management were key risk factors	~14
Virgilio et al. [27]	Median: 65 (58-76); N=50; gender not specified	Diabetes mellitus (58%); peripheral vascular disease (46%); recent antibiotic use (32%)	NR	Chronic wound colonization progressing to invasive infection by *Corynebacterium striatum*	Chronic wounds with poor healing; signs of localized infection (erythema, swelling); occasional systemic signs	Osteomyelitis in peripheral bones of lower extremities (21% of cases; N=11 patients)	Corynebacterium striatum	Wound colonization progressing to infection, particularly in immunocompromised or vascular-compromised patients	Primary antibiotic: Linezolid (used in 82% of cases). Other antibiotics: Vancomycin, daptomycin, beta-lactams when resistance or intolerance occurred. Duration: Median 6 weeks	Debridement performed in 78% of cases; amputation required in severe osteomyelitis cases	Poor healing in 46% of patients; treatment success varied significantly; multidrug-resistant *Corynebacterium *was a key factor in poorer outcomes	12
Rizvi et al. [28]	Median: 32 (immunocompetent) and 67 (immunocompromised); N=576; gender not specified	Malignancy (78.57% of immunocompromised cases); diabetes mellitus (21.43%)	86.54% immunocompetent; 13.46% immunocompromised	Nosocomial, post-surgical, or device-related opportunistic infections	Pain, swelling, erythema, purulent discharge, tenderness at the infection site, and non-healing surgical wounds	Osteomyelitis associated with orthopedic surgical sites, prosthetic joints, and open fractures	*Corynebacterium jeikeium* (most common), *Corynebacterium striatum*, *Corynebacterium minutissimum*, *Corynebacterium amycolatum*, *Corynebacterium urealyticum*, and others	Post-surgical contamination, hematogenous spread, or colonization of implanted devices	For *Corynebacterium jeikeium*: Linezolid most effective; vancomycin and teicoplanin used but some resistance observed. Other species: Sensitive to vancomycin, linezolid, chloramphenicol, tetracycline. Duration: NR	Device removal, debridement, and surgical site management performed in most cases	Antibiotic resistance was a major challenge, especially with *Corynebacterium jeikeium* and *Corynebacterium* *striatum*; outcomes varied with pathogen and treatment adequacy	NR
Tocco et al. [29]	Mean: 66 (29-84); N=70; 52 males, 18 females	Comorbidities included diabetes mellitus; obesity; chronic obstructive pulmonary disease; preoperative renal failure; peripheral arterial disease; hypertension	NR	Chronic post-surgical infections from open-heart surgery (post-sternotomy), associated with infected wounds or dehiscence	Sternal fistulas; chronic wound dehiscence; purulent drainage; radiological evidence of osteomyelitis	Sternum (post-sternotomy)	Coagulase-negative *Staphylococcus *spp. (60% of *Staphylococcus *spp.), *Staphylococcus aureus*, *Corynebacterium *spp. (10%), and others	Post-surgical wound contamination or unresolved infections from previous surgeries	Antibiotics: Linezolid effective for *Corynebacterium *spp.; prolonged therapy needed. Preferred regimens: Rifampicin-based combinations; teicoplanin, TMP-SMX, fusidic acid, clindamycin also used. Duration: 3-30 months (mean 7±4 months)	Conservative surgeries (wire removal and debridement) in 25 patients; no surgery in 45 patients	Healing achieved in 67 out of 70 patients; relapses occurred in 6 patients, requiring extended antibiotic therapy	12
Johani et al. [30]	Mean: not provided; N=20; gender not specified	All patients had diabetes mellitus; some had chronic wounds and prior antibiotic exposure	Immunocompromised	Chronic diabetic foot ulcers progressing to contiguous osteomyelitis	Chronic wounds with purulent drainage; exposed bone; erythema; localized pain; radiological findings of osteomyelitis	Small bones of the feet in diabetic patients	Polymicrobial infections including *Corynebacterium *spp. (*Corynebacterium amycolatum*, *Corynebacterium striatum*, *Corynebacterium tuberculostearicum*, *Corynebacterium jeikeium*), *Staphylococcus *spp., *Finegoldia *spp., *Streptococcus *spp., *Porphyromonas *spp., and *Anaerococcus *spp.	Antibiotic regimens not specified; emphasis on prolonged therapy due to biofilm formation	Antibiotic regimens were not explicitly detailed; study emphasized prolonged therapy due to biofilms	All patients underwent surgical debridement or amputation for diagnosis and treatment of infected bone	Biofilms identified in 80% of bone samples; polymicrobial infections in 70% of cases; treatment resistance due to biofilms was a major issue	NR
Trivedi and Merchant [31]	Median: 61 (48-87); N=7; 5 males, 2 females	Diabetes mellitus; neuropathy; vascular insufficiency; chronic kidney disease; malignancies (e.g., hepatocellular carcinoma)	Immunocompromised	Opportunistic infection with contiguous spread from soft tissue infections or direct inoculation during surgery	Chronic wounds with purulent drainage; non-healing ulcers; exposed bone; systemic signs like fever and lethargy	Multiple sites including the sternoclavicular joint, calcaneus, transmetatarsal stump, and great toe	*Corynebacterium striatum*; polymicrobial infections included *Staphylococcus *spp., *Enterobacter *spp., and *Pseudomonas aeruginosa*	Antibiotics used: Vancomycin, linezolid, ciprofloxacin, metronidazole, ertapenem, meropenem. Duration: Typically 4-6 weeks; initial IV therapy followed by oral therapy in some cases	Antibiotics: Vancomycin, linezolid, ciprofloxacin, metronidazole, ertapenem, meropenem. Duration: Typically 4–6 weeks (IV followed by oral therapy in some cases)	All patients underwent surgical interventions, including debridement, partial calcanectomy, and amputation	Cure in all cases; defined as granulation of the infected wound and resolution of clinical symptoms at follow-up	1-2

The ability of *C. minutissimum *to invade bone tissue may be attributed to its capacity to penetrate human osteoblasts. A study involving 13 *Corynebacterium *strains, including *C. minutissimum*, demonstrated their ability to invade osteoblasts, suggesting a mechanism for establishing chronic bone infections [1]. This intracellular survival may contribute to the chronicity and treatment challenges associated with these infections. Diagnosing *Corynebacterium *spp. infections poses significant challenges due to their rarity and the organisms' slow growth characteristics. In several cases from our review, initial cultures showed slow or scant growth, leading to delays in definitive identification. For example, Krish et al. reported that *C. xerosis *osteomyelitis was only identified after extended culture periods [22]. This delay underscores the importance of obtaining intraoperative samples and considering prolonged incubation periods when atypical organisms are suspected. Early and accurate identification is crucial for guiding targeted antimicrobial therapy and improving patient outcomes.

The antimicrobial susceptibility of *Corynebacterium *species varies, highlighting the need for susceptibility testing. In our analysis, vancomycin and linezolid were the most commonly used antibiotics, especially in cases involving multidrug-resistant strains like *C. jeikeium *and *C. striatum*. Overall, the literature indicates susceptibility to penicillin, vancomycin, linezolid, daptomycin, and other agents [6,7]. Dalal et al. reported the greatest susceptibility to vancomycin [6], while resistance to beta-lactams has been observed in some *Corynebacterium* species [1]. Bone and joint infections caused by *Corynebacterium *species are challenging to treat due to factors like antimicrobial resistance and biofilm formation; patients often require prolonged antimicrobial therapy and may need surgical intervention [3,32].

In this case, the patient was initially treated empirically with intravenous cefazolin targeting common pathogens like *Staphylococcus aureus*. Upon receiving susceptibility results identifying *C. minutissimum*, the antibiotic regimen was switched to oral doxycycline. This choice was appropriate due to several factors. Doxycycline has extensive coverage against gram-positive organisms, including *C. minutissimum*, and some gram-negative bacteria. It possesses good penetration into bone tissue, essential for treating osteomyelitis [33]. Furthermore, the literature supports the use of oral antibiotics with good bioavailability and bone penetration in managing osteomyelitis caused by *Corynebacterium *species. For instance, Virgilio et al. found that linezolid, an oral antibiotic with excellent bioavailability, was effective in treating osteomyelitis caused by *C. striatum *in diabetic patients [27]. Sasaki et al. reported successful treatment of *Corynebacterium *osteomyelitis with daptomycin after initial therapy with linezolid [9]. The oral route and long half-life of doxycycline facilitate ease of administration and improve adherence, as it can be dosed once or twice daily [34]. Additionally, using an oral antibiotic reduces the need for prolonged intravenous access, decreasing the risk of line-associated complications. The patient's clinical improvement following the initiation of doxycycline supports its effectiveness in treating *C. minutissimum *osteomyelitis. At follow-up, there were no signs of ongoing infection or hardware complications, indicating successful eradication of the pathogen.

This case contributes to the limited but growing body of literature on *C. minutissimum *as a causative agent in osteomyelitis and underscores several important clinical considerations. Clinicians should maintain a heightened suspicion for atypical pathogens like *C. minutissimum *in patients with chronic osteomyelitis, especially those with a history of trauma and orthopedic hardware implantation. Our literature review, encompassing 797 patient cases, emphasizes the importance of considering *Corynebacterium *species in the differential diagnosis of osteomyelitis, particularly in cases unresponsive to standard treatments [1,22,27]. Adequate tissue sampling during surgery is crucial for isolating uncommon organisms that may not be detected through standard culture techniques [19,25,30]. Extended incubation periods and advanced microbiological methods may improve detection rates, as suggested by the delayed identifications reported in multiple studies [20,22,30]. Empirical antibiotic regimens may need adjustment based on culture and sensitivity results to effectively target resistant or unusual pathogens. When feasible, oral antibiotics with good bioavailability and bone penetration, such as doxycycline, can be effective and improve patient compliance [12,14,21,27]. Given the challenges associated with biofilm formation and antibiotic resistance in *Corynebacterium *osteomyelitis, a multidisciplinary approach involving infectious disease specialists, microbiologists, and orthopedic surgeons is essential for optimal management.

## Conclusions

This case contributes to the growing body of literature on *C. minutissimum *as a rare but significant cause of osteomyelitis, particularly in patients with orthopedic hardware. Our literature review highlights that *Corynebacterium *species are being increasingly identified as causative agents in osteomyelitis, especially in patients who have prior surgical interventions, trauma, or immunocompromised states. The successful treatment of the patient in this case study with targeted oral doxycycline therapy, guided by antibiotic susceptibility testing, underscores the value of precise microbial identification and customized antimicrobial therapy in managing atypical bone infections. This case emphasizes the critical role of thorough intraoperative microbial sampling, including the use of extended incubation periods and advanced microbiological methods, to identify uncommon pathogens that standard methods might overlook. By sharing this case and the insights from our literature review, we aim to raise clinician awareness of *C. minutissimum *and other *Corynebacterium *species as potential causes of osteomyelitis and encourage prompt, targeted interventions to improve patient outcomes.
